# Workflow to improve patient recruitment for clinical trials within hospital information systems – a case-study

**DOI:** 10.1186/1745-6215-9-2

**Published:** 2008-01-11

**Authors:** Martin Dugas, Matthias Lange, Wolfgang E Berdel, Carsten Müller-Tidow

**Affiliations:** 1 Department of Medical Informatics and Biomathematics, University of Münster, Domagkstrasse 9, 48149 Münster, Germany; 2 IT Centre, Universitätsklinikum Münster, Domagkstrasse 3, 48149 Münster, Germany; 3 Department of Medicine A, Hematology and Oncology, Universitätsklinikum Münster, Domagkstrasse 3, 48149 Münster, Germany

## Abstract

**Background:**

The identification of suitable patients is a common problem in clinical trials that is especially evident in tertiary care hospitals.

**Methods:**

We developed and analysed a workflow, which uses routine data captured during patient care in a hospital information system (HIS), to identify potential trial subjects. Study nurses or physicians are notified automatically by email and verify eligibility.

**Results:**

As a case study we implemented the system for acute myeloid leukemia (AML) trials in Münster. During a test period of 50 days 41 patients were identified by the system. 13 could be included as new trial patients, 7 were already included during earlier visits. According to review of paper records no AML trial patient was missed by the system. In addition, the hospital information system further allowed to preselect patients for specific trials based on their disease status and individual characteristics.

**Conclusion:**

Routine HIS data can be used to support patient recruitment for clinical trials by means of an automated notification workflow.

## Background

Patient recruitment is crucial for the success of any clinical trial. Complete identification of eligible patients ensures both timely execution of the trial and avoids selection bias. Recruitment is a common and relevant issue in clinical trials. A recent analysis of more than 100 trials showed that less than a third of the trials achieved their original recruitment target and half were awarded an extension [1].

Given the high patient turnover of modern hospitals in conjunction with complex inclusion and exclusion criteria of many simultaneous active trials, patient recruitment can be a challenging task. For instance, the registry of the national cancer institute contains more than 5000 active clinical trials [2]. There are several systems to support identification of a suitable clinical trial for a specific patient [3,4]. However, these systems are usually not integrated into regular patient care. The treating physicians are often not aware about the possibility of a clinical trial in a specific situation. The wide range of available trials and the heavy workload of physicians might limit the capacity to identify trials that are relevant for the patient.

**Figure 1:**
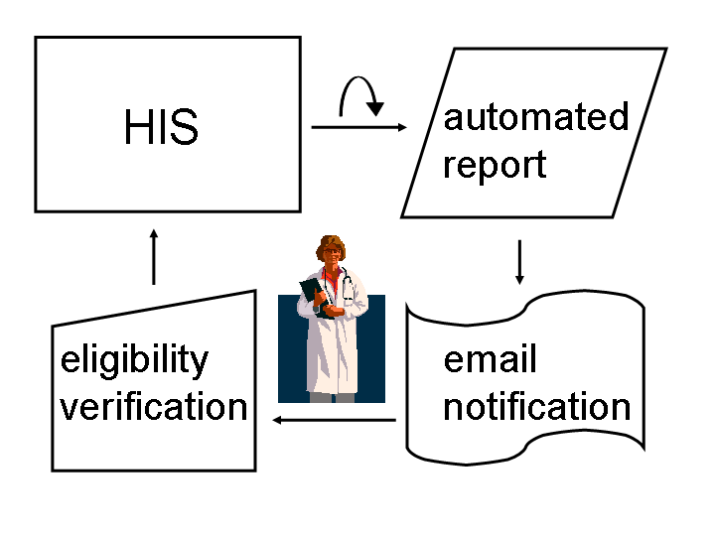
**Overview of HIS-based patient recruitment**. An automated HIS report triggers email notification of study nurses and physicians, who verify patient eligibility for a specific clinical trial.

In this paper we present and analyse a workflow, which uses routine data captured during patient care in a major hospital information system (HIS), to identify possible trial subjects. There are few reports of this strategy, mainly in the outpatient setting [5-7]. The key differentiator of our approach is to use coded diagnosis information, which is available in the inpatient setting, to improve precision – and thereby acceptance – of electronic alerts. Routine HIS data does usually not contain all information regarding inclusion and exclusion criteria. Therefore we aim at an automated screening method. Study nurses or physicians are notified if a patient would be suitable for clinical trials. Eligibility is verified by using the resources of the data management system. This approach avoids an unnecessary burden of additional data gathering in patients unlikely to be suitable for a specific trial. Figure 1 presents the overall workflow.

In the next sections we describe how this workflow can be implemented within a standard hospital information system and present preliminary data from a pilot study in our hospital.

## Methods

**Figure 2:**
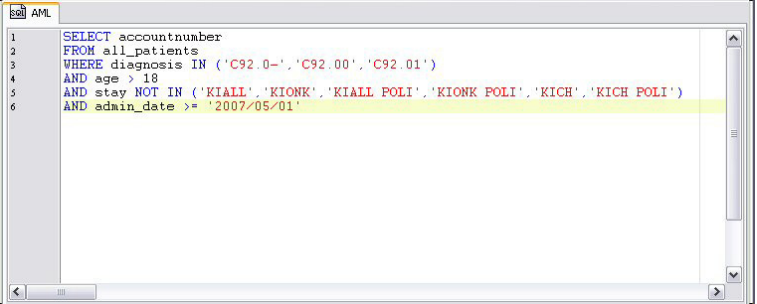
**Sample database query**. Adult patients with AML are identified.

For each clinical trial a database query is generated using the report generator of the HIS (ORBIS^® ^from Agfa Healthcare) [8]. Depending on inclusion and exclusion criteria of each trial and available HIS items, the query is designed to provide high recall and precision. Typically, admission diagnosis (primary as well as secondary diagnoses, coded according to international classification of diseases), patient age, patient gender and routine lab values can be employed in this query. Figure 2 presents a sample query for a clinical trial on AML.

**Figure 3:**
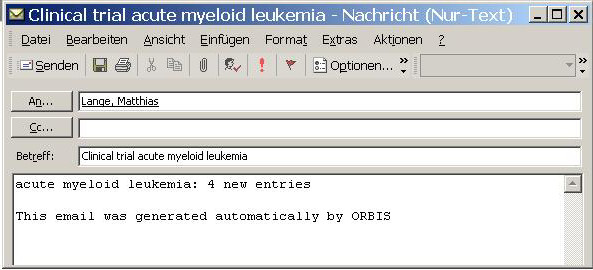
Screen shot of automatic notification email.

This query is executed regularly (for instance once per day) and its output is compared to a table of previous query results. If there are new potential trial subjects, an email to the responsible study nurses and/or physicians is generated. To address privacy regulations, this email does not contain any patient name. Instead, it just states that there are potential new patients for a certain trial (Figure 3).

**Figure 4:**
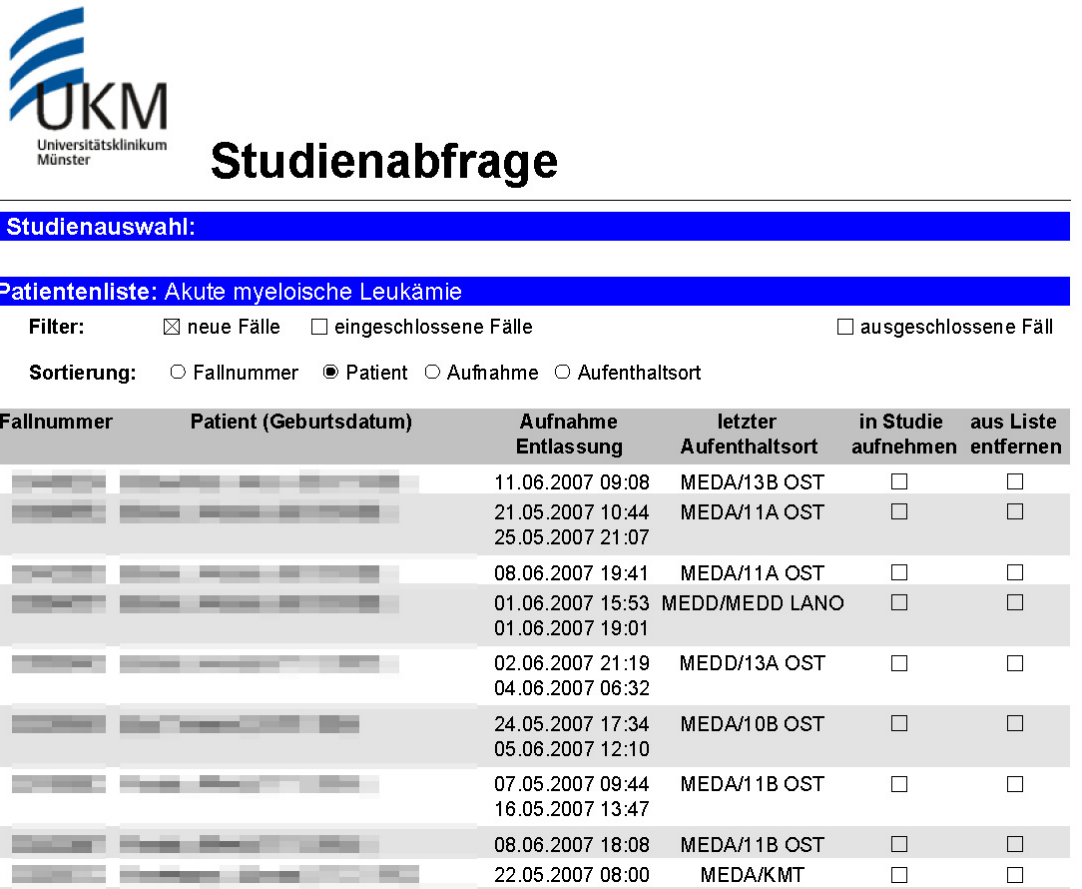
**AML trial report with patient list (in German)**. It can be filtered by new cases ("neue Fälle"), included ("eingeschlossene") and excluded ("ausgeschlossene") cases and sorted by case number ("Fallnummer"), patient, admission date ("Aufnahme") and location ("Aufenthaltsort"). For each patient case number, name, date of birth, date of admission and discharge as well as location are provided. For each case inclusion ("in Studie aufnehmen") or exclusion ("aus Liste entfernen") can be documented.

This notification email requests to log on to the HIS, where the authorized user can retrieve the report of potential trial patients (Figure 4). Access to HIS requires direct involvement in the care for a specific patient. By full access to the electronic patient record additional information can be retrieved to verify eligibility. If the patient is eligible for the trial, a study physician contacts the patient to obtain informed consent. By means of a custom HIS form actual inclusion or exclusion can be documented for each patient.

Given the large number of simultaneously active trials, technical parameters for each trial are organized by a trial management tool. It provides trial title, data query for each trial, contact persons for email notification and references.

The time to train users to use the system was tracked by the investigator.

## Results

Our HIS-based patient recruitment workflow was implemented for ongoing Münster AML trials (publications from completed Münster AML trials see [9,10]). All patients with AML were screened for trial eligibility. During a test period of 50 days (starting May 2007) 41 patients were identified by the system. 13 could be included as new trial patients, 7 were already included during earlier visits. During these 50 days altogether 363 inpatients were admitted to the department of hematology and oncology. Review of paper records showed that no AML trial patient was missed by the system.

From a technical perspective, the system remained stable, CPU load of reporting was small. According to comments of study nurses, the notification system eased access to potential patients and reduced the number of phone calls and site visits to identify suitable patients. In addition, the hospital information system further allowed to preselect patients for specific trials based on their disease status and individual characteristics.

One session of user training (approximately 1 hour) for study nurses and physicians was sufficient. Currently, the system is rolled out for additional clinical trials in neurology and dermatology.

## Discussion

Clinical trials are characterized by highly specialized documentation needs, specifically tailored to the requirements of an individual trial. In the context of Good Clinical Practice (GCP) [11] regulations, data monitoring needs to be applied to achieve high data quality. It is necessary to validate electronic documentation systems for clinical trials. General hospital information systems are – at least at present – not capable to address all documentation needs of clinical trials [12].

However, during the evolvement process of electronic patient records more and more clinical information becomes available in HIS. In some cases – for instance laboratory values – electronic data becomes a primary data source. Our approach utilises routine HIS data – captured for administrative and clinical purposes – as an automated screening tool for patient recruitment. Even if not all items needed for inclusion and exclusion are available in the routine HIS, basic information like diagnosis, gender and age appear to be sufficient for an automated screening tool.

There are some reports in the literature regarding HIS-based support for patient recruitment. For instance, Embi [5] describes a system for a diabetes mellitus trial in an outpatient setting which resulted in a significant increase of patient enrollment. Weiner [6] analysed a real-time alerting system in the emergency department and observed improved study investigator notification. Afrin [7] reports mass screening of lab values for a lupus nephritis trial, which also highlights the issue of alert precision: 7 Mio. lab values were screened, 70 potential patients identified, only 3 were enrolled into the trial.

Many false positive alerts can be annoying for physicians and impact user acceptance. In our case, 20 of 41 alerts were related to trial patients and no AML trial patient was missed. A test period of 50 days is certainly not long enough for a valid assessment of improved recruitment, therefore we plan to do this kind of analysis after about one or two years of routine operation. However, it demonstrates a relatively high precision of notifications. We capitalised on a specific, coded diagnosis which is commonly entered into electronic systems after patient encounter. This delayed documentation is acceptable for inpatient alerts, but obviously limits real-time notification in the outpatient setting.

From our perspective, integration into standard HIS is relevant for several reasons: A direct link to the patient record is provided which facilitates eligibility verification. Since all diagnoses and suspected diagnoses are entered into the system at the day of admission (inpatient or outpatient), eligible patients can routinely be identified before start of therapy. Also, redundant data entry is avoided, because routine HIS data is re-used for trial purposes.

Data privacy regulations are covered by standard HIS access controls and protocols, so there is no need for additional authentication systems. Clinical users are familiar with the HIS which limits the need for additional training activities. When informed consent is obtained, additional orders can be placed in the HIS order-entry-system according to each trial protocol.

From a technical perspective, our approach does not need external systems and avoids firewall issues. On the other hand, it requires vendor-specific technical configuration for each trial at each hospital site and is therefore primarily suited for clinical trials with few centers.

## Conclusion

Routine HIS data can be used to support patient recruitment for clinical trials by means of an automated notification workflow.

Our workflow is not a replacement for electronic data capture (EDC) systems in clinical trials, it is targeted to support patient recruitment. True integration of clinical and research documentation by connecting HIS and EDC systems still remains an ambitious, but important goal for future systems.

## Abbreviations

AML: Acute myeloid leukemia;

EDC: Electronic data capture;

GCP: Good Clinical Practice;

HIS: Hospital Information System.

## Competing interests

The author(s) declare that they have no competing interests.

## Authors' contributions

MD designed the concept and wrote the manuscript. ML designed the software. WB and CMT contributed clinical requirements and reviewed the manuscript. All authors read and approved the final manuscript.
